# Identification of NAC Transcription Factors Associated with Leaf Senescence in *Clerodendrum japonicum*

**DOI:** 10.3390/ijms26188846

**Published:** 2025-09-11

**Authors:** Congcong Wang, Guihua Liao, Yu Duan, Lingye Su, Chunmei He, Mingfeng Xu, Hongfeng Wang

**Affiliations:** Guangdong Academy of Forestry, Guangdong Provincial Key Laboratory of Silviculture, Protection and Utilization, Guangzhou 510520, China

**Keywords:** *Clerodendrum japonicum*, leaf senescence, transcriptome, WGCNA, NAC transcription factor, gene co-expression network

## Abstract

Leaf senescence, the terminal phase of leaf development, is governed by transcription factor (TF)-mediated genetic reprogramming events that significantly impact plant physiology and productivity. While TF-mediated senescence regulation has been demonstrated in various plant species, the underlying molecular mechanisms remain incompletely understood. This study investigated the regulatory roles of NAC family TFs in leaf senescence using two *Clerodendrum japonicum* lines exhibiting contrasting senescence phenotypes. Through integrated transcriptome sequencing, weighted gene co-expression network analysis (WGCNA), and functional enrichment approaches, we systematically analyzed temporal gene expression patterns during leaf senescence. Phenotypic characterization revealed distinct chlorophyll degradation dynamics between the lines, quantified by SPAD values. Transcriptomic analysis identified 232 consistently differentially expressed genes (DEGs) across senescence stages, among which 193 were annotated as NAC transcription factors. WGCNA delineated senescence-associated gene modules, with the turquoise and darkred modules showing particularly strong correlations with senescence progression. Further investigation identified 25 NAC genes exhibiting stage-specific expression patterns, and functional analysis revealed that 15 of these were significantly enriched in organ senescence-related pathways. qRT-PCR validation confirmed that the four core NAC regulators showed up to 5-fold higher expression in the early-senescing line during late senescence stages. These findings delineate the NAC-mediated regulatory network governing leaf senescence in *C. japonicum*, offering potential molecular targets for manipulating senescence progression, which warrants further functional characterization and practical application in plant improvement.

## 1. Introduction

Leaf senescence is a fundamental biological process crucial for plant development and environmental adaptation [1,2]. As the final stage of leaf development, senescence involves the highly coordinated breakdown of cellular components and nutrient recycling, processes essential for plant survival and reproductive success [1,3,4,5]. In agricultural and horticultural systems, understanding leaf senescence is particularly valuable as it directly affects yield, quality, and ornamental value, with premature senescence leading to significant economic losses [2]. While considerable progress has been made in model species like Arabidopsis and rice [6,7], the molecular mechanisms governing senescence in ornamental plants, especially woody ornamentals, remain poorly understood, creating an important knowledge gap in plant biology research [8]. Elucidating these mechanisms is essential for improving plant productivity, stress resilience, and longevity.

NAC (NAM, ATAF1/2, and CUC2) transcription factors constitute one of the largest plant-specific TF families and are master regulators of stress responses, development, and senescence [9,10]. Their functional diversity is exemplified in species such as Arabidopsis, where AtORE1/NAC092 promotes senescence via a miR164-mediated pathway [11], and AtNAP/OsNAP directly regulates chlorophyll catabolic genes like SGR [12]. Beyond model species, NAC TFs play equally critical roles: ZmNAC132 integrates ABA and jasmonate signaling to coordinate leaf senescence in maize [13], while MdNAC047 modulates ethylene-mediated salt tolerance in apple [14,15]. Similarly, NACs regulate both natural and induced senescence in Petunia [16] and ethylene-mediated cell expansion in rose petals [17]. Despite these advances, NAC-mediated senescence regulation in non-model woody ornamentals remains poorly understood, representing a significant knowledge gap.

*Clerodendrum japonicum*, a perennial ornamental shrub exhibiting natural variation in senescence timing among cultivars, presents an ideal system to address this gap. High-throughput sequencing and network biology, particularly Weighted Gene Co-expression Network Analysis (WGCNA), enable the resolution of temporal regulatory dynamics often missed in single time point comparisons [18,19]. Recent advances in high-throughput sequencing and network biology have revolutionized our understanding of transcriptional regulation during leaf senescence [20]. In this study, we investigated the molecular mechanisms of leaf senescence in *C. japonicum* by comparing late-senescing ‘Pink Flower’ (P) and early-senescing ‘Red Flower’ (R) lines. To capture the dynamic transcriptional changes underlying this process, we employed transcriptome sequencing across multiple time points coupled with Weighted Gene Co-expression Network Analysis (WGCNA). WGCNA is a powerful tool for resolving temporal dynamics of gene regulation and identifying stage-specific transcriptional modules that single time point comparisons often miss [21,22,23]. While WGCNA has been widely applied in model crops (e.g., tomato) [24,25], its utility in non-model woody ornamentals like *C. japonicum* remains unexplored. Here, we bridge this gap by integrating WGCNA with temporal transcriptomics to dissect NAC-mediated senescence regulation in this species.

While widely applied in model crops, its utility in non-model woody ornamentals like *C. japonicum* remains unexplored. We identified key NAC transcription factors regulating senescence timing, characterized their expression patterns, and revealed conservation and divergence in senescence regulation between these contrasting lines. These findings establish *C. japonicum* as a valuable model for studying senescence in woody ornamentals and provide potential molecular targets for manipulating senescence in horticultural species.

## 2. Results

### 2.1. Phenotypic Characterization of Leaf Senescence in Early- and Late-Senescing Lines

**Figure 1:**
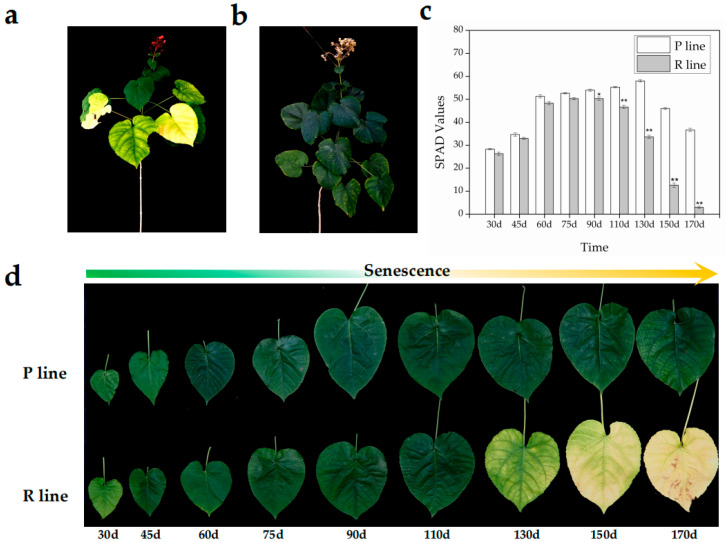
Leaf senescence phenotypes in early- (R) and late-senescing (P) *Clerodendrum japonicum* lines. (**a**) Whole plant phenotype of the early-senescing line (R) at day 170 after first leaf sprouting. (**b**) Whole plant phenotype of the late-senescing line (P) at day 170 after first leaf sprouting. (**c**) SPAD values of leaves at different developmental stages for lines R and P. Vertical bars represent SE (*n* = 3); asterisk (*) indicates significant difference (*p* ≤ 0.05), double asterisk (**) indicates highly significant difference (*p* ≤ 0.01) by Student’s *t*-test. (**d**) Representative leaf senescence phenotypes of lines R and P at different developmental stages.

The senescence process in the two *Clerodendrum japonicum* lines was monitored by measuring leaf SPAD values (indicative of chlorophyll content) at different time points. The lines exhibited distinct leaf senescence phenotypes during growth (Figure 1a,b). The ‘Pink Flower’ line (P), classified as late-senescing, maintained notably high SPAD values throughout the measurement period, indicating a slow rate of chlorophyll degradation. In contrast, the ‘Red Flower’ line (R) showed a rapid decrease in SPAD values during the latter half of the measurement period, signifying accelerated senescence (Figure 1c). Visual phenotypes at day 170 clearly illustrated the advanced senescence in the R line compared to the P line (Figure 1d).

### 2.2. Transcriptome Sequencing and Data Processing

To investigate differential gene expression mechanisms during leaf aging, we performed RNA-Seq analysis across nine developmental stages. To quantify gene expression, Illumina short-read sequencing was performed. Raw sequencing reads from 54 cDNA libraries (18 samples: 9 time points × 2 lines, with triplicates) were rigorously filtered, yielding 319.54 GB of high-quality clean data (≥5.92 GB per sample). The reads were aligned to a PacBio-derived reference transcriptome (see Section 4). All samples exhibited Q30 scores > 93.42% (Table S1), confirming data suitability for downstream analysis. Using transcript variants obtained from long-read sequencing (PacBio) sequencing as a reference (Sequel II system with SMRT Link v10.1), alignment and quantitative analysis using RSEM (v1.3.3) showed that among the genes identified in the PacBio Iso-Seq data, at least 76.4% of the genes were consistently detected in the short-read (Illumina, NovaSeq X series with software v1.3) sequencing dataset (Table S2). Approximately 2.12 billion valid reads were generated, with over 96.92% (2.07 billion reads) successfully mapped to the reference database (Table S3), supporting the feasibility of subsequent analyses.

**Figure 2:**
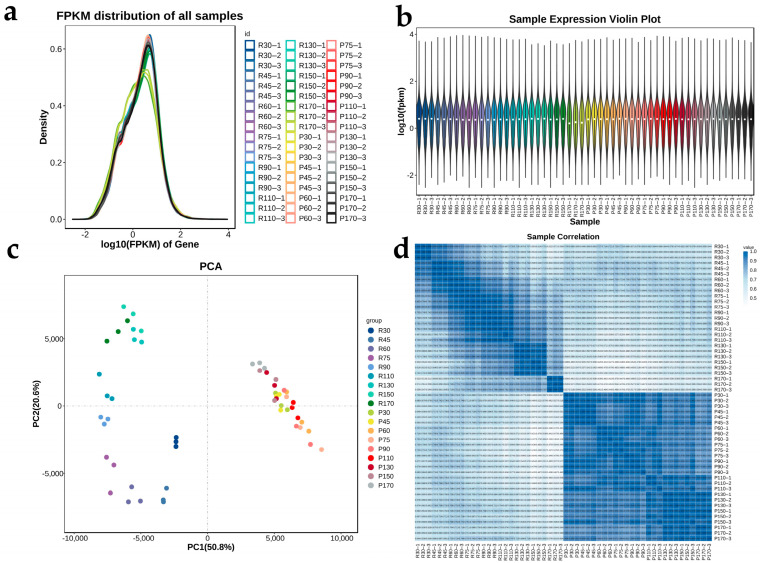
Transcriptome data quality assessment and sample relationships. (**a**) Expression density plots across samples. (**b**) Violin plots of gene expression profiles. Groups: R (Red Flower, early-senescing), P (Pink Flower, late-senescing); numbers indicate days. (**c**) Principal Component Analysis (PCA) plot based on gene expression levels. (**d**) Heatmap of Pearson correlation coefficients between samples.

Expression density plots based on FPKM values visualized gene expression distribution across samples (Figure 2a). Violin plots indicated high reproducibility among biological replicates within each treatment group and a normal distribution of data without apparent outliers (Figure 2b). While comparable trends were observed among most groups, the R-170 group displayed a distinct distribution, suggesting substantial gene expression alterations during late-stage senescence. Principal Component Analysis (PCA) revealed clear separation between the R and P line groups, with no overlap between lines. Within the R line, pronounced dispersion occurred among different time points, whereas the P line treatments showed relatively minor separation (Figure 2c). The initial PCA was also used to assess potential batch effects arising from library preparation and sequencing dates; no significant batch effects were observed. As a precautionary measure, any minor technical variation was corrected using the removeBatchEffect function from the limma R package (v3.58.1) prior to all downstream analyses. High Pearson correlation coefficients among biological replicates within each group, visualized in a heatmap, further confirmed reproducibility (Figure 2d).

### 2.3. Differential Gene Expression and Functional Enrichment

**Figure 3:**
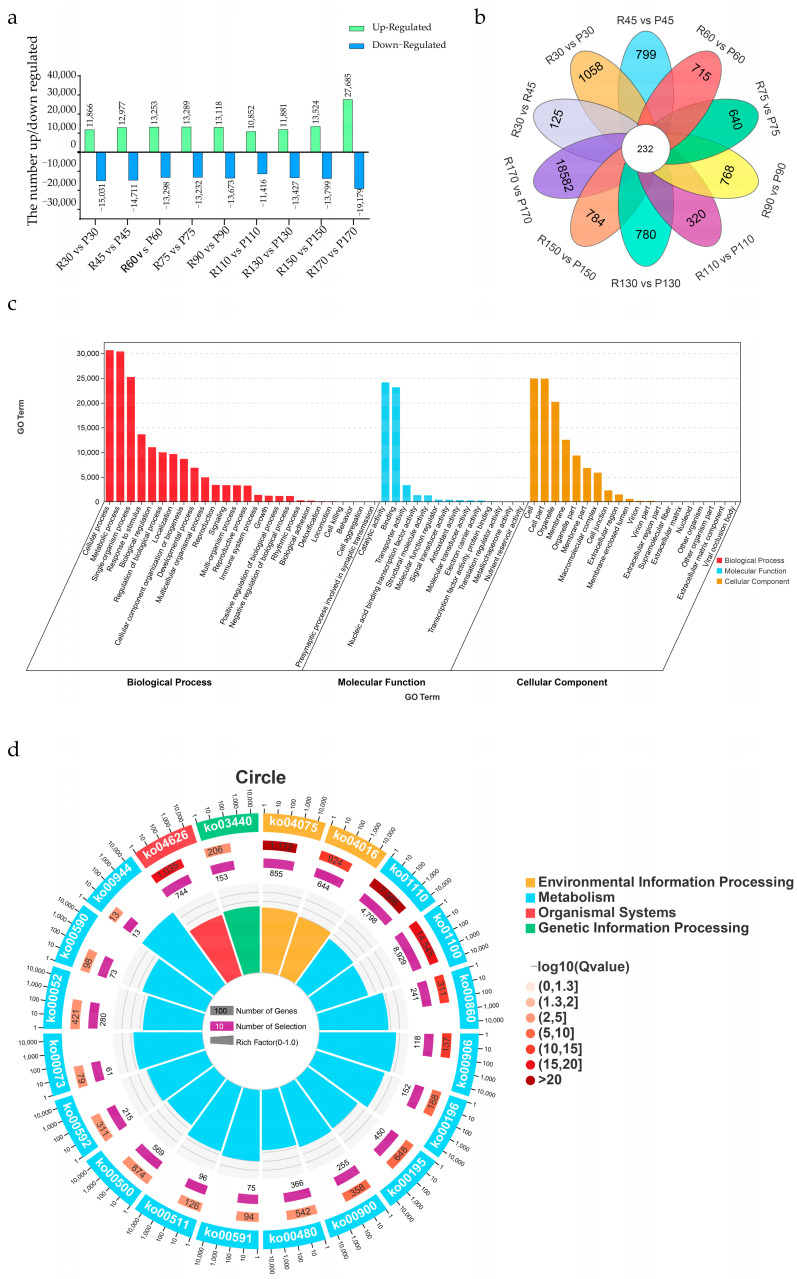
Differential gene expression analysis during leaf senescence. (**a**) Number of up- and downregulated DEGs at each time point comparison between lines R and P. (**b**) Venn diagram showing the overlap of DEGs across all nine time point comparisons, identifying 232 consistently differentially expressed genes. (**c**) GO enrichment analysis of DEGs (BP: Biological Process, MF: Molecular Function, CC: Cellular Component). (**d**) KEGG pathway enrichment analysis of DEGs.

Differentially expressed genes (DEGs) between the P and R lines at each time point were identified using DESeq2 (v1.22.2) (significance thresholds: |log2FoldChange| ≥ 1 and FDR < 0.05). The number of DEGs varied during senescence progression, peaking in 170-day-old leaves (Figure 3a). A Venn diagram comparing DEGs across all nine time points (R30-vs-P30 to R170-vs-P170) identified 232 genes consistently differentially expressed throughout leaf development and senescence (Figure 3b). Functional categorization of DEGs identified across the nine stages via Gene Ontology (GO) enrichment revealed their involvement in three domains: Biological Processes (BP), Molecular Functions (MF), and Cellular Components (CC) (Figure 3c). In BP, DEGs were predominantly associated with “cellular process” and “metabolic process”. In MF, “catalytic activity” and “binding” were most enriched. In CC, “cell” and “cell part” were predominant. Kyoto Encyclopedia of Genes and Genomes (KEGG) analysis revealed significant enrichment in pathways related to “Metabolism”, “Environmental Information Processing”, “Genetic Information Processing”, and “Organismal Systems” (Figure 3d). This suggests leaf senescence involves coordinated metabolic reprogramming (e.g., carbohydrate, amino acid, lipid metabolism for resource redistribution), environmental sensing (e.g., light, temperature, stress signaling like MAPK cascades), precise transcriptional/translational control, and systemic coordination (e.g., hormone signaling, immune responses linking senescence to hormonal homeostasis and stress adaptation).

Although leaf phenotypes appear similar between lines P and R during early and middle developmental stages, the differentially expressed genes (DEGs) identified in these stages likely play crucial roles in several key biological processes. These DEGs may be involved in metabolic reprogramming, such as carbohydrate, amino acid, and lipid metabolism, which are essential for leaf growth and development and lay the foundation for subsequent senescence. Additionally, DEGs may participate in environmental sensing and signaling pathways, such as MAPK cascades, helping plants adapt to environmental changes and maintain leaf health during early and middle developmental stages. They may also play roles in precise transcriptional and translational control, ensuring accurate and coordinated gene expression within cells. Furthermore, DEGs may contribute to systemic coordination through the regulation of hormone signaling and immune responses, which are vital for overall plant physiology during these stages.

### 2.4. Identification and Stage-Specific Expression Analysis of NAC Transcription Factors

**Figure 4:**
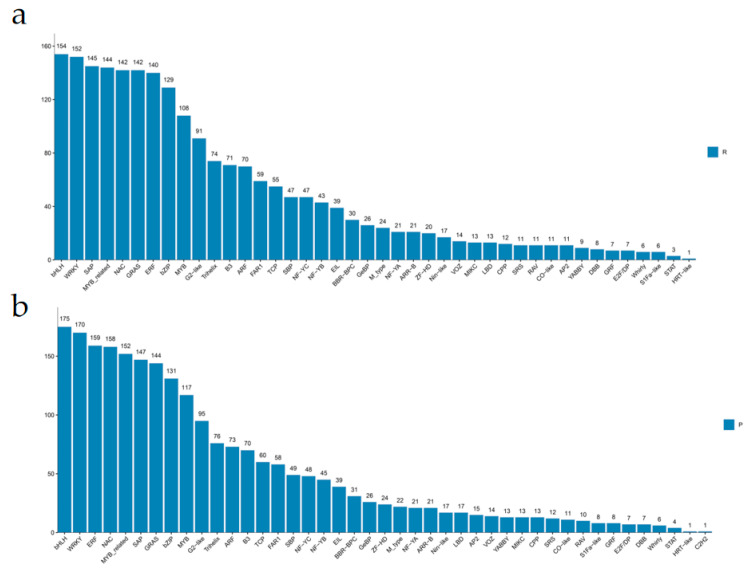
Classification of predicted transcription factors (TFs) in *Clerodendrum japonicum* lines R and P. (**a**) Classification and abundance of transcription factor families in the early-senescing R line. (**b**) Classification and abundance of transcription factor families in the late-senescing P line. (Table S4 provides detailed counts). Groups: R (Red Flower, early-senescing), P (Pink Flower, late-senescing).

Using PlantTFDB (v5.0), we predicted 2155 and 2289 putative TFs in the R and P lines, respectively, classifying them into families (Figure 4a,b and Table S4). The R line contained 142 NAC genes, while the P line contained 158. NAC genes in both lines were clustered into 5 distinct expression trend modules (Figure S1). In the P line, cluster 1 had the most genes (*n* = 47) and cluster 4 the least (*n* = 13). In the R line, cluster 1 also had the most (*n* = 29) and cluster 5 the least (*n* = 15) (Table S5). The expression of senescence-associated NAC genes exhibited minimal variation during early developmental stages (30–60 days), with differential expression becoming evident upon senescence initiation (90–110 days) and demonstrating substantial alterations during advanced senescence (130–170 days). Focus was placed on clusters showing upregulation in mid-to-late senescence. Given the accelerated senescence phenotype of the R line, NAC gene clusters with progressively upregulated patterns (clusters 2, 3, and 5) were selected for further analysis. Among the 232 consistently DEGs identified across all senescence stages (Figure 3b), bioinformatic analysis revealed 193 (83.2%) encoded NAC-domain transcription factors. Notably, this set of NAC genes constituted the vast majority (83.2%) of all consistently differentially expressed transcription factors, highlighting the exceptional specificity of this family in the senescence process of *C. japonicum*. This striking predominance suggests NAC family members play disproportionate roles in coordinating senescence progression in *C. japonicum*.

### 2.5. Identification of Gene Modules Coexpressed in Association with Leaf Senescence

**Figure 5:**
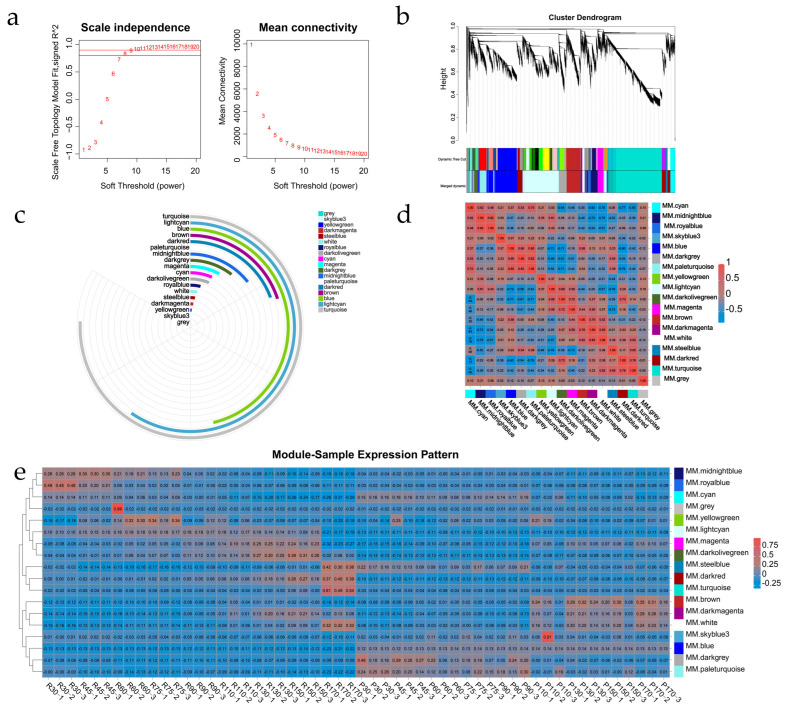
Weighted Gene Co-expression Network Analysis (WGCNA) of leaf senescence. (**a**) Determination of soft-thresholding power (β = 20). (**b**) Hierarchical clustering dendrogram of genes with module colors (after merging). (**c**) Distribution of gene counts across the 18 modules. (**d**) Module-module eigengene correlation heatmap. (**e**) Module eigengene expression patterns across all samples. R: Red Flower line (early-senescing), P: Pink Flower line (late-senescing); numbers indicate days.

To gain a network-level understanding of leaf senescence, we performed Weighted Gene Co-expression Network Analysis (WGCNA) (Figure 5). The pickSoftThreshold function determined an optimal soft-thresholding power of 20 (scale-free topology fit R^2^ > 0.8; Figure 5a). Hierarchical clustering and dynamic tree cutting identified initial modules, which were subsequently merged based on eigengene similarity, resulting in 18 consensus modules (Figure 5b). The turquoise and lightcyan modules contained the majority of transcripts (Figure 5c). Module-module relationship analysis (based on eigengene correlation) revealed the strongest correlations between turquoise and darkred modules, and between lightcyan and darkolivegreen modules (Figure 5d). Stage-specific expression dynamics showed significant upregulation of the turquoise and darkred modules specifically during late-stage senescence (170 days) in the R line (R170 samples), but not in the P line (P170 samples) (Figure 5e). Pearson correlation analysis between module membership (MM) and gene significance (GS) for senescence (correlated with SPAD) identified the darkred (cor = 0.67, *p* < 0.05) and turquoise (cor = 0.66, *p* < 0.05) modules as highly senescence-associated, containing 7824 genes collectively. Using stringent thresholds (GS > 0.6 and |MM| > 0.9), 1582 hub genes were identified in turquoise and 283 in darkred (Figure S2, Tables S6 and S7).

### 2.6. Functional Analysis of NAC Genes Associated with Senescence Stages

**Figure 6:**
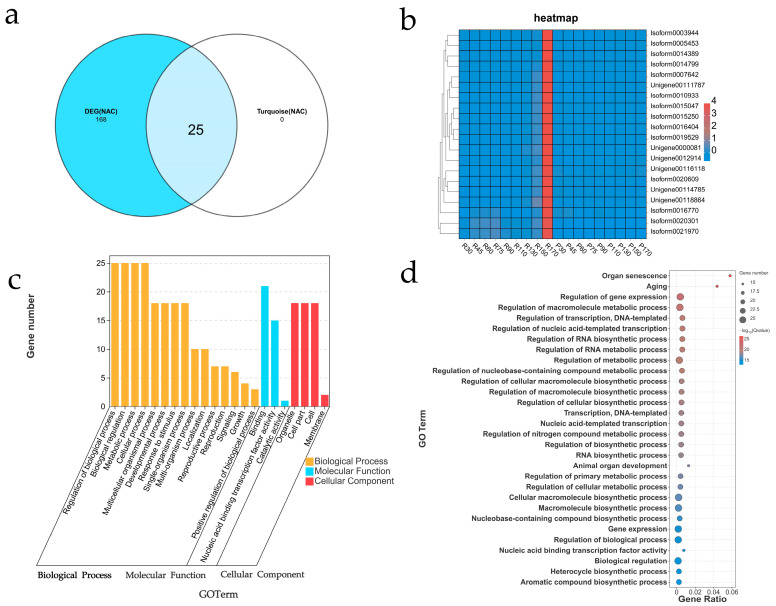
Identification and functional analysis of senescence-associated NAC transcription factors. (**a**) Venn diagram identifying target NAC genes from two screening approaches: Turquoise Module Hubs (GS > 0.6 and |MM| > 0.9), DEGs (Union across time points), Consistently Expressed DEGs (232 genes). (**b**) Heatmap of expression patterns for the 25 target NAC genes across all developmental stages in lines R and P. (**c**) GO enrichment (Biological Process domain) for the 25 target NAC genes. (**d**) Enrichment analysis of specific GO terms for the 25 target NAC genes. Top enriched terms shown.

To refine the NAC gene set associated with senescence, we intersected the 193 NAC DEGs (from Section 2.3) with the 25 NAC hub genes in the turquoise module (GS > 0.6 and |MM| > 0.9), yielding 15 NACs linked to organ senescence (GO:0010260). Screening the turquoise module (GS > 0.6 and |MM| > 0.9) identified 25 NAC genes; no qualifying NAC genes were found in darkred (Tables S6 and S7). From the union of DEGs across all pairwise time point comparisons (both lines), 193 NAC genes were identified (Section 2.3, Figure 3a and Table S8). Venn diagram analysis of these two NAC gene sets identified the target NAC cohort (Figure 6a). Expression patterns of the 25 target NAC genes (Table S8) across developmental stages in both lines revealed distinct trends: In the early-senescing R line, all hub NAC genes showed gradual upregulation as senescence progressed. Conversely, in the late-senescing P line, hub NAC genes exhibited diverse patterns: three clusters showed overall downregulation, six clusters showed initial downregulation followed by upregulation, and one cluster showed upregulation (Figure S3). A heatmap confirmed these contrasting expression trends between lines (Figure 6b). Gene Ontology (GO) analysis of the 25 target NACs revealed primary enrichment in “Regulation of biological process” (GO:0050789), “Biological regulation” (GO:0065007), “Metabolic process” (GO:0008152), and “Cellular process” (GO:0009987) within the Biological Process domain (Figure 6c, Table S9). Enrichment analysis of specific GO terms identified “organ senescence” (GO:0010260), “aging” (GO:0007568), and “regulation of gene expression” (GO:0010468) as the most significantly enriched (Figure 6d). Strikingly, 15 of these NAC hubs (60%) were functionally annotated to organ senescence (GO:0010260, FDR < 0.05), representing a significant enrichment compared to their proportion among all NAC DEGs (7.8%, 15/193) (Table S8).

### 2.7. Selection and Experimental Verification of Key NAC Genes

**Figure 7:**
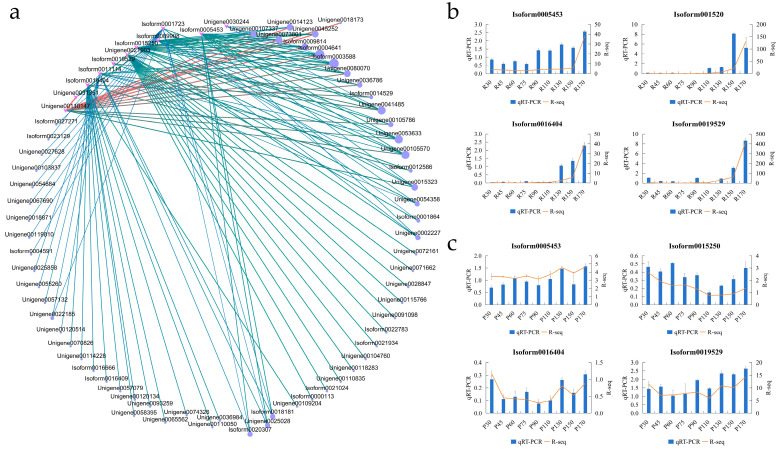
Identification of key NAC transcription factors and validation of transcriptome data. (**a**) Top 10 most connected transcription factors in the turquoise module from WGCNA. Six *NAC* genes (*Isoform0016404, Isoform0019529, Unigene0021903, Isoform0015250, Isoform0005453, Unigene0031351*) are highlighted among the highly connected TFs. (**b**) qRT-PCR validation of the expression patterns of six randomly selected genes across all nine time points (30, 45, 60, 75, 90, 110, 130, 150, and 170 days) in the R line. Values are mean ± SE (*n* = 3). The expression trends validate those observed in the RNA-Seq analysis. (**c**) qRT-PCR validation of the expression patterns of six randomly selected genes across all nine time points (30, 45, 60, 75, 90, 110, 130, 150, and 170 days) in the P line. Values are mean ± SE (*n* = 3). The expression trends validate those observed in the RNA-Seq analysis.

WGCNA identified six NAC genes among the top 10 most connected TFs in the turquoise module (Isoform0016404, Isoform0019529, Unigene0021903, Isoform0015250, Isoform0005453, Unigene0031351) (Figure 7a). GO enrichment identified 15 NACs associated with organ senescence (e.g., Isoform0003944 (NAC078), Isoform0005453 (NAC078), Isoform0019529 (NAP1), Isoform0016404 (NAP2), Unigene00116118 (NAP2), Isoform0015250 (JA2L)) (Section 2.6). Intersecting these two candidate sets yielded four core NAC genes: Isoform0005453 (NAC078), Isoform0015250 (JA2L), Isoform0016404 (NAP2), and Isoform0019529 (NAP1). To validate the temporal expression patterns from the transcriptome data, quantitative real-time PCR (qRT-PCR) was performed on randomly selected DEGs across all nine developmental time points (30–170 days). The results confirmed the reliability of the RNA-Seq data (Figure 7b,c).

## 3. Discussion

### 3.1. NAC Transcription Factors as Central Regulators of Senescence

NAC transcription factors are established master regulators of leaf senescence across plant species. Our integrated analysis in *C. japonicum* not only reinforces this conserved role but also identifies specific NAC members that underpin natural variation in senescence timing. The contrasting chlorophyll degradation dynamics between the R and P lines (Figure 1) are a phenotypic manifestation of divergent transcriptional programs, at the heart of which lie four core NAC TFs: NAC078, NAP1, NAP2, and JA2L. The distinct expression patterns of these regulators-specifically, the progressive upregulation in the early-senescing R line contrasted with the delayed or attenuated induction in the P line-strongly suggest that they act as critical pacemakers setting the tempo of senescence. The 20-day delay in senescence associated with modulated NAP1/2 expression in the P line highlights the significant physiological impact of fine-tuning the expression of these NAC genes, positioning them as prime targets for manipulating longevity in ornamental species. These findings underscore delayed senescence as a valuable horticultural trait that prolongs visual appeal and extends the period of carbon assimilation, consistent with previous reports linking delayed senescence to sustained photosynthetic activity and enhanced stress tolerance [26].

An interesting observation is the temporal discrepancy between the onset of physiological senescence (evident as declining chlorophyll levels at 130 and 150 days in the R line, Figure 1c) and the massive transcriptional reprogramming (observed at 170 days, Figure 3a and Figure 6b). We propose several non-mutually exclusive explanations for this lag. First, the initial chlorophyll degradation may be triggered by post-translational activation of existing enzyme pools (e.g., through phytohormone signaling or redox changes) rather than de novo transcription. The early, subtle expression changes in key regulators (e.g., the initial upregulation of NAP1/2 between 90 and 110 days, Figure 6b and Figure S3) might be sufficient to initiate this process. Second, the massive transcriptional shift at 170 days likely represents a later, amplifying ‘feed-forward’ phase, where early activated TFs (like NACs) strongly induce the expression of a vast suite of downstream catabolic genes, committing the cell to irreversible senescence. Finally, it is possible that the critical transcriptional changes occur in a specific, small subset of cells (e.g., initiating at the leaf tip or vasculature) early on, and our bulk RNA-seq approach averaged this signal with that of non-senescing cells, diluting its magnitude until the process became widespread throughout the leaf at 170 days. This phenomenon underscores the complex, multi-layered regulation of leaf senescence.

### 3.2. WGCNA Reveals Senescence-Associated Gene Modules

Transcriptomic profiling identified 232 consistently differentially expressed genes (DEGs), among which NAC TFs were significantly enriched. WGCNA resolved temporal regulatory networks and identified the turquoise module as strongly correlated with senescence progression, particularly the late-stage upregulation observed in the R line. Within this module, 25 NAC genes were identified as hub genes, 15 of which were significantly associated with the gene ontology term “organ senescence” (GO:0010260), reinforcing the role of NAC TFs as master regulators of senescence [27,28,29]. The four core NACs, exhibiting high network connectivity and GO association with “organ senescence”, “chlorophyll catabolic process”, and “response to ethylene”, emerged as key regulators. Their distinct, progressive upregulation in the R line, contrasting with nuanced dynamics (e.g., initial downregulation followed by late upregulation of NAP1/2) in the P line, implicates them as positive regulators analogous to ANAC092/AtNAP and OsNAP [30,31]. This mirrors conserved NAC-dependent programs across angiosperms, yet the P line’s dynamics suggest quantitative modulation of NAC dosage, rather than simple activation, fine-tunes senescence timing-supported by graded SlNAP reductions delaying senescence in tomato [32].

### 3.3. Functional and Evolutionary Implications

Functional enrichment (GO/KEGG) linked DEGs to core senescence processes: metabolic reprogramming (e.g., carbohydrate, amino acid metabolism), environmental sensing/signaling (e.g., MAPK cascades), transcriptional/translational control, and systemic hormone/stress coordination (Figure 3c,d), resonating with mechanisms in model species [33,34,35], The co-expression of JA2L-an orthologue of *Arabidopsis* JUNGBRUNNEN1 integrating jasmonate and ABA cues-suggests multi-hormone signals feed into the NAC hub [36]. By analogy to ANAC092 trans-activating chlorophyll catabolism genes [37], early/sustained NAP1/2 induction in R likely accelerates pigment breakdown, while their transient repression in P may permit prolonged photosynthate accumulation. The P line’s delayed phenotype may involve early repression of NACs postponing catabolism, followed by compensatory late upregulation.

Although the leaf phenotypes of the P and R lines were largely indistinguishable during early and middle development (Figure 1d), significant transcriptional differences were already present (Figure 3a). We hypothesize that these early-stage DEGs are not direct effectors of senescence but rather constitute a preparatory transcriptional landscape that primes the leaves for their subsequent divergent senescence trajectories. Many of these early DEGs were enriched in pathways related to stress response, hormone signaling (e.g., ABA and jasmonate), and secondary metabolism (Figure 3c,d). This suggests that the early-senescing R line may be in a state of heightened alertness or metabolic predisposition, making it more susceptible to the internal and external cues that trigger senescence. Conversely, the late-senescing P line may maintain a more robust homeostasis or delayed metabolic shift, effectively buffering it against these signals and delaying the onset of catabolic processes. Thus, the difference in senescence timing may be less about the activation of a unique senescence program and more about the failure to maintain longevity mechanisms, with the early DEGs representing the molecular underpinnings of this differential priming.

The evolutionary conservation of these core NAC regulators underscores their potential as universal targets for senescence manipulation. Our candidate genes have clear functional homologs with well-established roles in senescence across diverse plant species. Specifically, NAP1 (Isoform0019529) and NAP2 (Isoform0016404) are orthologs of Arabidopsis AtNAP and rice OsNAP [30], which are central regulators of leaf and organ senescence that directly activate chlorophyll catabolic and senescence-associated genes. Similarly, NAC078 (Isoform0005453) is a homolog of Arabidopsis AtNAC078, which has been implicated in age-dependent leaf senescence. The presence of these conserved regulators in *C. japonicum* suggests that the core transcriptional circuitry governing senescence is deeply evolutionarily conserved among angiosperms. This high degree of conservation greatly enhances the translational potential of our findings, as strategies aimed at modulating the activity of these NAC genes in *C. japonicum* are likely to be applicable to a broad range of horticultural and crop species for extending longevity and improving stress resilience.

### 3.4. Comparative Perspective on Senescence in Woody Plants

Placing our findings in a comparative context with well-studied woody models like Populus and Acer highlights both conserved and unique aspects of senescence in *C. japonicum*. Similarly to Populus [8], NAC transcription factors appear to be master regulators of senescence in *C. japonicum*, underscoring a conserved regulatory role for this family in perennial species. However, *C. japonicum* exhibits a unique feature: the presence of stable, genetically determined early- and late-senescing lines within the same species. This natural variation provides a powerful, physiologically relevant system to dissect the regulatory mechanisms controlling senescence timing without the confounding effects of exogenous stress or artificial induction. Furthermore, while many Populus NACs are stress-induced, the core NAC regulators we identified (e.g., NAP1, NAP2) in *C. japonicum* are strongly associated with developmental senescence, suggesting a more specialized role in the age-dependent program. The distinct expression dynamics of these NACs-particularly the delayed induction in the late-senescing line-point to a potential mechanism for fine-tuning the pace of senescence that may be unique to or particularly pronounced in this species. This comparative perspective not only reinforces the central role of NACs but also positions *C. japonicum* as an ideal model for studying the natural genetic variation underlying senescence timing in woody ornamentals.

### 3.5. FLimitations and Future Directions

The conservation of NAC TFs as senescence regulators from Arabidopsis and rice to maize and now *C. japonicum* underscores deep evolutionary conservation of core modules [38,39]. While the core NAC module is conserved, lineage-specific expansion and functional divergence have been reported in non-model species. However, lineage-specific NAC expansion/diversification and observations like novel petunia NAC pathways [40,41] highlight potential species-specific adaptations. Our findings exhibit this duality: conserved NAC involvement alongside unique expression dynamics (e.g., specific P line patterns), emphasizing the importance of studying non-model woody species to understand senescence plasticity and evolution. Manipulating senescence timing has significant horticultural implications. Delaying senescence enhances esthetic value in ornamentals like *C. japonicum* and can optimize yield/resource allocation in crops [42,43]. The identified core NAC genes provide prime targets for genetic (e.g., CRISPR-Cas9 knockout/overexpression) or chemical manipulation strategies.

While this study establishes a comprehensive transcriptomic framework, functional validation of the core NAC genes via stable transformation or virus-induced gene editing is imperative. WGCNA identifies correlation, not causality. Furthermore, our bulk leaf transcriptome data could mask cellular heterogeneity; single-cell or spatially resolved profiling may reveal if NAC expression heterogeneity contributes to the gradual SPAD decline in P leaves. Integrating proteomics and metabolomics (e.g., quantifying ABA, jasmonate) is essential to elucidate protein-level regulation and hormone-NAC feedback loops [44,45,46]. Finally, field trials under diverse environmental stresses are needed to assess the practical relevance for improving plant performance.

## 4. Materials and Methods

### 4.1. Plant Material and Phenotyping

Plants of the R (‘Red Flower’, early-senescing) and P (‘Pink Flower’, late-senescing) lines of *Clerodendrum japonicum* were cultivated at the Guangdong Academy of Forestry Sciences nursery (23°11′59″ N, 113°22′11″ E) in a 3:2:1 (*v*/*v*/*v*) peat/yellow clay/river sand substrate under uniform irrigation and fertilization. Three-year-old plants were used. Chlorophyll content was estimated non-destructively using SPAD values (SPAD-502 meter, Minolta, Tokyo, Japan) [11,12]. SPAD measurements were taken on the first-round sprouted leaves at 30, 45, 60, 75, 90, 110, 130, 150, and 170 days after sprouting (*n* = 3 biological replicates per time point per line). On day 170, leaf samples corresponding to the SPAD measurement points were collected [47], immediately flash-frozen in liquid nitrogen, and stored at −80 °C for RNA extraction.

### 4.2. RNA Extraction, Library Construction, and Sequencing

Total RNA was extracted from all leaf samples using the TaKaRa MiniBEST Universal RNA Extraction Kit (Takara Bio, Beijing, China). RNA concentration was measured using a NanoDrop^®^ spectrophotometer (Thermo Fisher Scientific, Waltham, MA, USA), and integrity was assessed using an Agilent^®^ 2100 Bioanalyzer (Agilent Technologies, Santa Clara, CA, USA). Only RNA samples with an RNA Integrity Number (RIN) ≥ 7.0 were used for subsequent library construction. High-quality RNA was reverse-transcribed into first-strand cDNA using the Clontech SMARTer PCR cDNA Synthesis Kit (Takara Bio, Beijing, China). Double-stranded cDNA was synthesized via PCR amplification, purified with AMPure PB Beads (Pacific Biosciences, Menlo Park, CA, USA), and used to construct SMRTbell libraries. Library quality was assessed, and qualified libraries were sequenced. Raw sequencing data were processed in SMRT Link v6.0 (Pacific Biosciences, Menlo Park, CA, USA) for read classification, annotation, and sequence characterization [48]. For full-length transcriptome sequencing, total RNA from a pooled sample was used to construct a SMRTbell library. Library quality was assessed, and qualified libraries were sequenced on the PacBio Sequel II platform to generate long reads for reference transcriptome assembly. Raw sequencing data were processed in SMRT Link v6.0 (Pacific Biosciences) for read classification, annotation, and characterization. For quantitative RNA-seq analysis, total RNA from each of the 54 individual samples was used for cDNA library construction. Libraries were prepared using the NEBNext^®^ Ultra™ RNA Library Prep Kit (New England Biolabs, Ipswich, MA, USA) for Illumina^®^ and sequenced on an Illumina NovaSeq 6000 platform to generate 150 bp paired-end reads for gene expression quantification.

### 4.3. Transcriptome Data Processing and Differential Expression Analysis

Raw reads were quality-filtered using fastp with parameters: adapter trimming, removal of reads with >10% Ns, removal of poly-A reads, and discarding reads where >50% of bases had Q ≤ 20. The PacBio Iso-Seq data were assembled to generate a high-quality reference transcriptome (GSA accession number: CRA005391). Clean reads were aligned to the *Clerodendrum japonicum* full-length transcriptome reference (GSA accession number: CRA005391) using HISAT2 (v2.1.0). Gene expression levels were quantified as FPKM using RSEM/bowtie2 [49]. Differential expression analysis between R and P lines at each time point was performed using DESeq2 (v1.22.2) with a negative binomial model [50]. Genes with |log2 Fold Change| ≥ 1 and FDR < 0.05 were considered DEGs. To account for potential technical variation, batch effects were corrected using the removeBatchEffect function from the limma R package (v3.50.0) prior to differential expression analysis. Read counts were normalized internally during the DESeq2 analysis using the median-of-ratios method. In this study, we compiled a comprehensive list of gene IDs along with their corresponding Gene Ontology (GO) and Kyoto Encyclopedia of Genes and Genomes (KEGG) annotations (Table S11). Functional enrichment analysis of DEGs was performed using GO (http://geneontology.org accessed on 1 December 2024) and KEGG (http://www.kegg.jp accessed on 1 December 2024) databases, with Benjamini–Hochberg adjusted *p*-value < 0.05 considered significant.

### 4.4. Transcription Factor Identification

Transcription factors (TFs) were predicted using PlantTFDB (http://planttfdb.gao-lab.org/ accessed on 1 March 2025) [51]. HMMER 3.0 with Pfam domain profiles (v27.0) was used to detect DNA-binding domains [52]. Putative TFs were classified into families based on conserved domain architectures [53].

### 4.5. Weighted Gene Co-Expression Network Analysis (WGCNA)

WGCNA was performed using the WGCNA package (v1.72) in R [54]. The pickSoftThreshold function determined the optimal soft-thresholding power (β = 20) for a scale-free network (R^2^ > 0.8). Genes were clustered hierarchically, and modules were identified using dynamic tree cutting. Modules with highly correlated eigengenes (Pearson r > 0.75) were merged. Module eigengene expression patterns were analyzed across developmental stages. Gene significance (GS) for senescence was defined as the correlation between gene expression and SPAD values (negative correlation as senescence increases). Module membership (MM) represented the correlation of a gene’s expression with the module eigengene. Modules significantly correlated with GS (|cor| > 0.6, *p* < 0.05) were identified. Hub genes within these modules were filtered using GS > 0.6 and |MM| > 0.9.

### 4.6. Quantitative Real-Time PCR (qRT-PCR) Validation

Total RNA was isolated from leaf samples using Trizol reagent (Invitrogen; Waltham, MA, USA). Genomic DNA was removed, and cDNA was synthesized from 1 μg RNA using the PrimeScript™ RT reagent Kit with gDNA Eraser (TaKaRa; Beijing, China). Gene-specific primers were designed using Primer5 (Premier Biosoft; Palo Alto, CA, USA). qRT-PCR was performed in triplicate using TB Green™ Premix Ex Taq™ II (Tli RNaseH Plus) (TaKaRa; Beijing, China) on an ABI StepOne Plus system. Cycling conditions: 95 °C for 10 min; 40 cycles of 95 °C for 15 s, 60 °C for 35 s. Relative expression was calculated using the 2^−ΔΔCt^ method [55]. Primer sequences are listed in Supplementary Table S10.

## 5. Conclusions

In summary, our multi-omics approach has delineated a sophisticated NAC-mediated transcriptional network that governs the timing of leaf senescence in *Clerodendrum japonicum*. The discovery of four core NAC regulators (NAC078, JA2L, NAP2, and NAP1) as key determinants of phenotypic variation provides a mechanistic understanding of longevity in this ornamental species. Beyond fundamental insights, this study delivers directly applicable resources for molecular breeding. The identified NAC genes, particularly those with delayed expression in the late-senescing line, constitute powerful genetic tools for biotechnological interventions. We propose that modulating the expression of these candidates, via gene editing or marker-assisted selection, holds immediate promise for developing novel *C. japonicum* cultivars with extended ornamental display periods and enhanced resilience. This work thus establishes a framework for translating regulatory gene discovery into tangible improvements in woody ornamental breeding.

## Data Availability

The raw RNA-Seq data generated in this study have been deposited in the Genome Sequence Archive (GSA) at the China National Center for Bioinformation (CNCB) under BioProject accession number PRJCA018252. The data are accessible via the CNCB submission portal (https://ngdc.cncb.ac.cn/gsub/, accessed on 13 July 2023) under GSA accession number CRA011821.

## Supplementary Materials

The following supporting information can be downloaded at: https://www.mdpi.com/article/10.3390/ijms26188846/s1.

## Abbreviations

The following abbreviations are used in this manuscript: DEG, Differentially Expressed Gene; FDR, False Discovery Rate; FPKM, Fragments Per Kilobase per Million mapped reads; GO, Gene Ontology; KEGG, Kyoto Encyclopedia of Genes and Genomes; NAC, NAM, ATAF1/2, CUC2; PCA, Principal Component Analysis; qRT-PCR, Quantitative Real-Time PCR; SPAD, Soil–Plant Analysis Development; TF, Transcription Factor; WGCNA, Weighted Gene Co-expression Network Analysis; GSA, Genome Sequence Archive.
