# Supplementary figures and images for: Identification of NAC Transcription Factors Associated with Leaf Senescence in Clerodendrum japonicum
Source: Int J Mol Sci. 2025 Sep 11;26(18):8846. doi: 10.3390/ijms26188846 (PMC12470089; doi:10.3390/ijms26188846)

a

R line

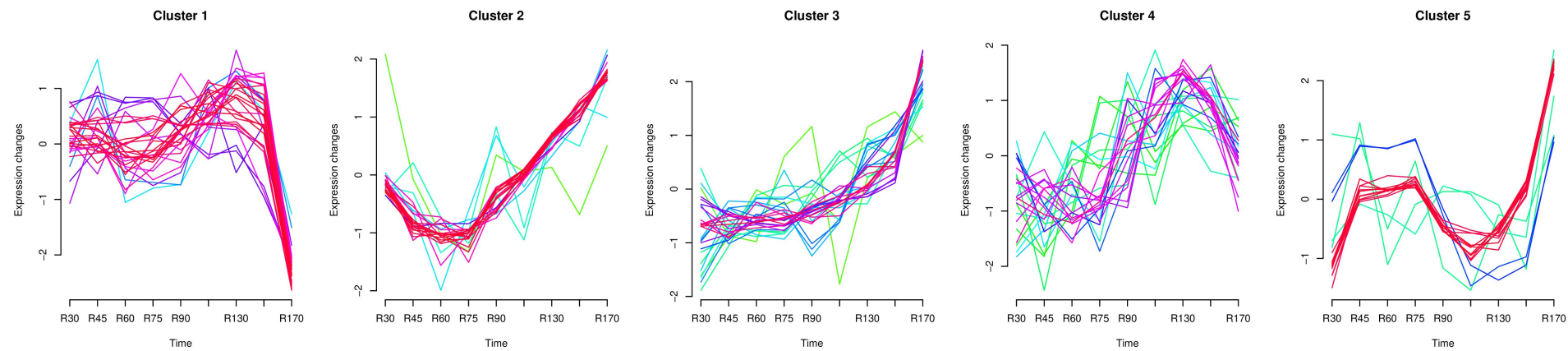

b

P line

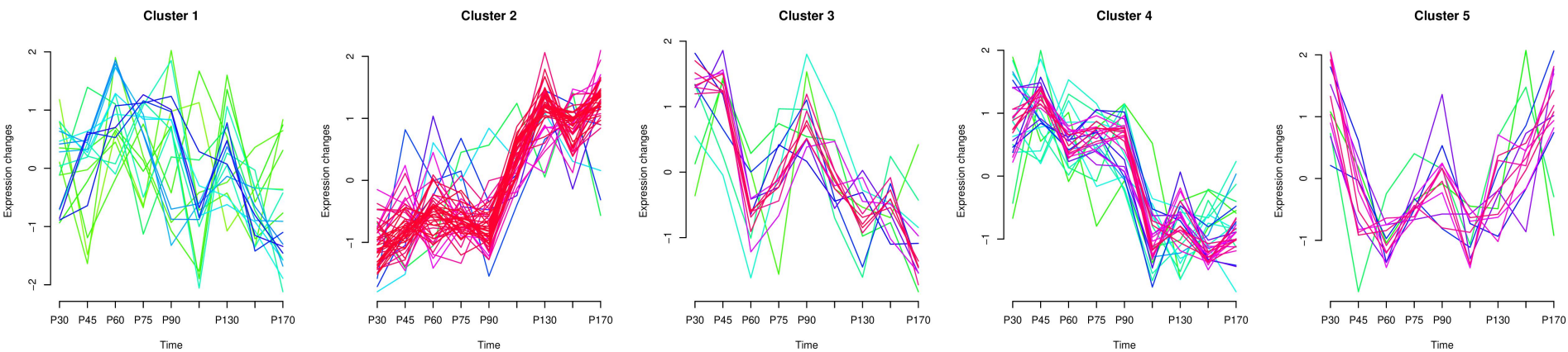

Supplement: Supplementary file 1 [file ijms-26-08846-s001.zip › figure S1-Cluster Analysis of NAC Gene Expression Patterns in R and P Lines.pdf]

a

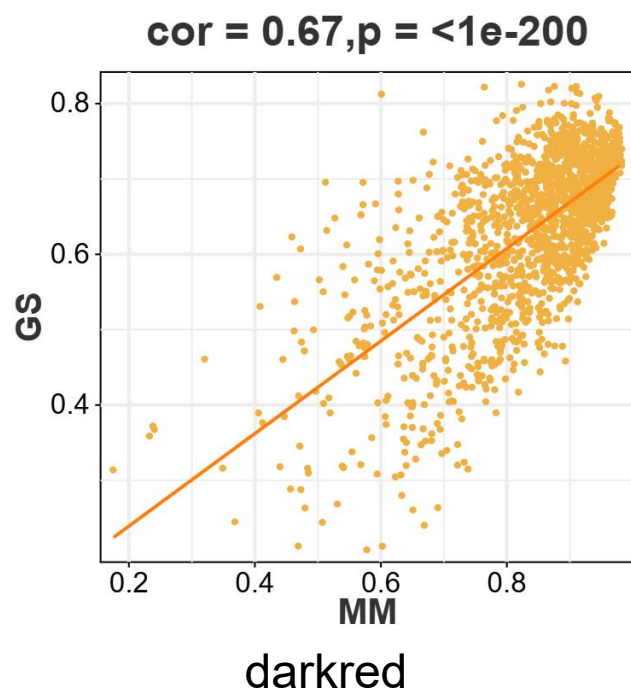

b

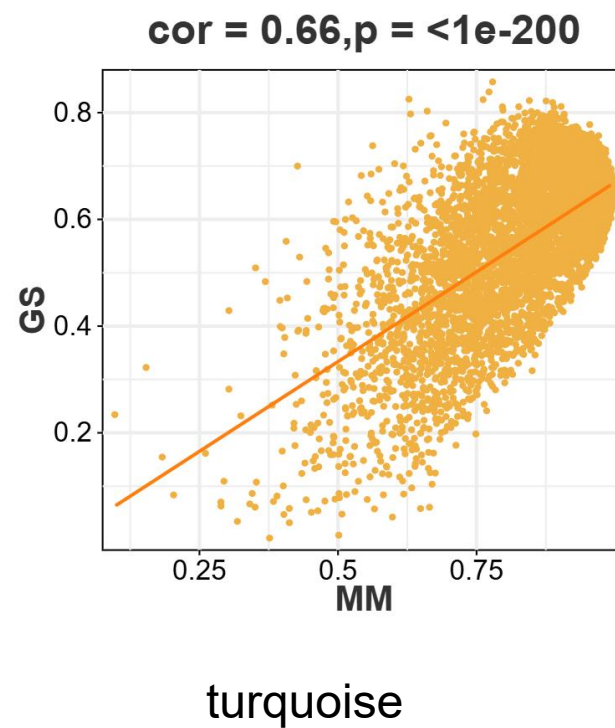

Supplement: Supplementary file 1 [file ijms-26-08846-s001.zip › figure S2-Pearson correlation analysis of module membership (MM) and gene significance (GS) for senescence.pdf]

P

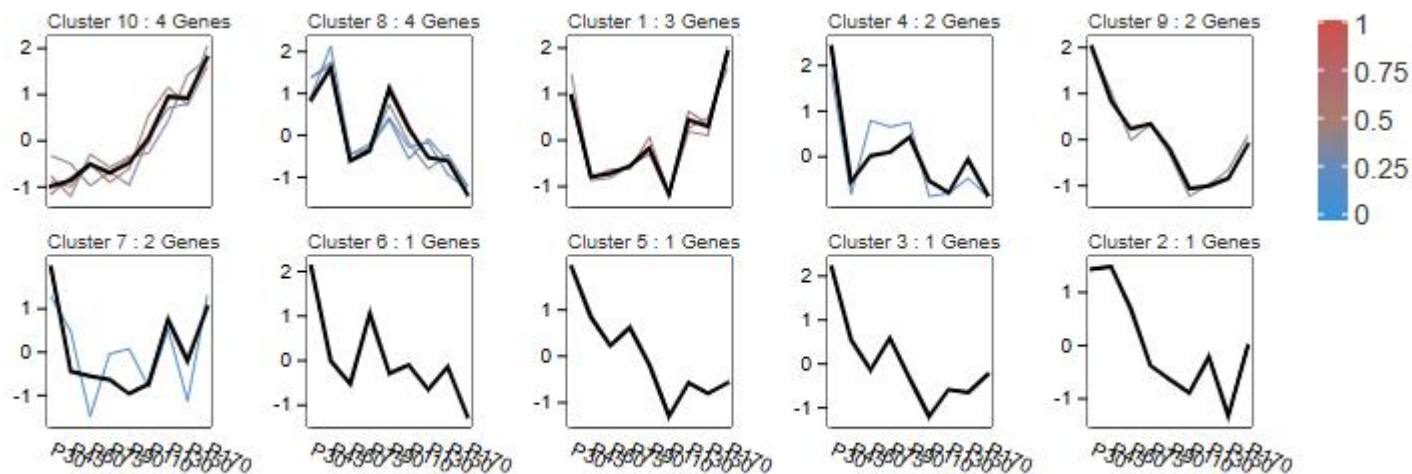

R

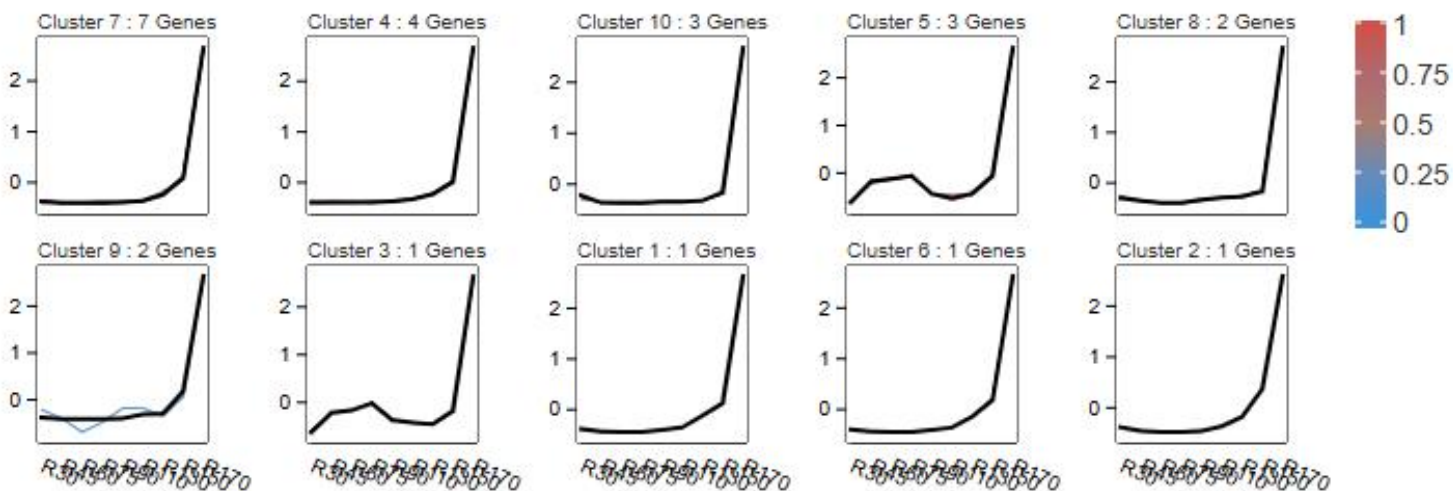

Supplement: Supplementary file 1 [file ijms-26-08846-s001.zip › figure S3-Expression dynamics of hub NAC genes during senescence progression in early-senescing (R line) and late-senescing (P line) genotypes..pdf]
